# Niraparib in pancreatic cancer with germline or somatic DNA damage repair (DDR) gene alterations (BRCA and beyond): a phase II study (NIRA-PANC)

**DOI:** 10.1093/oncolo/oyag225

**Published:** 2026-07-17

**Authors:** Anup Kasi, Raed Moh’d Taiseer Al-Rajabi, Joaquina Celebre Baranda, Anwaar Saeed, Junqiang Dai, Prabhakar Chalise, Scott Weir, Brent Sear, Erin Carroll, Shannon Bradbury, Alok Tripathi, Stephen Hyter, Venkatadri Beeki, Richard J McKittrick, Sean Kumer, Timothy Schmitt, Steven Soper, Andrew K Godwin, Weijing Sun

**Affiliations:** Department of Medical Oncology, University of Kansas Medical Center, Kansas City, KS 66160, United States; Department of Medical Oncology, University of Kansas Medical Center, Kansas City, KS 66160, United States; Department of Medical Oncology, University of Kansas Medical Center, Kansas City, KS 66160, United States; Department of Medical Oncology, University of Kansas Medical Center, Kansas City, KS 66160, United States; Department of Biostatistics and Data Science, University of Kansas Medical Center, Kansas City, KS 66160, United States; Department of Biostatistics and Data Science, University of Kansas Medical Center, Kansas City, KS 66160, United States; Department of Toxicology, Pharmacology and Therapeutics, University of Kansas Medical Center, Kansas City, KS 66160, United States; Department of Toxicology, Pharmacology and Therapeutics, University of Kansas Medical Center, Kansas City, KS 66160, United States; Department of Medical Oncology, University of Kansas Medical Center, Kansas City, KS 66160, United States; Department of Medical Oncology, University of Kansas Medical Center, Kansas City, KS 66160, United States; Department of Internal Medicine, University of Kansas Medical Center, Kansas City, KS 66160, United States; Department of Pathology and Laboratory Medicine, University of Kansas Medical Center, Kansas City, KS 66160, United States; Department of Medical Oncology, University of Kansas Medical Center, Kansas City, KS 66160, United States; Department of Medical Oncology, University of Kansas Medical Center, Kansas City, KS 66160, United States; Department of Surgery, University of Kansas Medical Center, Kansas City, KS 66160, United States; Department of Surgery, University of Kansas Medical Center, Kansas City, KS 66160, United States; School of Biomedical Engineering, University of Kansas Medical Center, Kansas City, KS 66160, United States; Department of Internal Medicine, University of Kansas Medical Center, Kansas City, KS 66160, United States; Department of Medical Oncology, University of Kansas Medical Center, Kansas City, KS 66160, United States

**Keywords:** pancreatic cancers, PARP inhibitor, niraparib, metastatic and unresectable pancreatic cancer, phase II trial, 6-month PFS, median PFS, median OS, BRCA1/2 mutation, ECOG

## Abstract

**Background:**

Globally, pancreatic cancer is one of the leading causes of cancer-related mortality. Although FDA-approved chemotherapy regimens are available, rapid deterioration is often observed after first-line treatment. The PARP inhibitor niraparib may offer a therapeutic benefit in patients with advanced pancreatic cancer, necessitating thorough investigation.

**Patients and methods:**

This was an open-label, single-arm, phase II trial involving 37 patients with metastatic or unresectable pancreatic cancer with DNA damage repair (DDR) gene alterations. Patients were administered niraparib (300 or 200 mg daily, based on weight and platelet count) in 28-day cycles until disease progression, unacceptable toxicity, or withdrawal. Efficacy was assessed using the 6-month progression-free survival (PFS) rate as the primary endpoint.

**Results:**

Of the 37 patients, 29 were evaluated for efficacy. The 6-month PFS rate was 41.38% (12/29 patients; 95% CI, 4.70%-100%), the median PFS was 4.4 months (95% CI, 3.6-6.5 months), and the median OS was 10.3 months (95% CI, 7.6-15.9 months). Further subgroup analysis revealed that the BRCA1/2 germline mutation-positive patient group (*n* = 14) reported a 6-month PFS rate of 50% and a median overall survival (mOS) of 12.1 months, while the non-BRCA group (*n* = 15) showed a 6-month PFS rate of 33.33% and a mOS of 10.3 months. Adverse events occurred in 78% of patients, the most common being anemia (27%), and no treatment-related deaths were observed.

**Conclusions:**

These data demonstrate clinical activity of niraparib in patients with metastatic or unresectable pancreatic cancers harboring DDR gene defects. Future studies are warranted to establish their roles in diverse genetic patient subpopulations.

**ClinicalTrials.gov identifier:**

NCT03553004.

Lessons learned

- In this phase II open-label study, we investigated the potential therapeutic efficacy of niraparib, a PARP inhibitor, in patients with metastatic or unresectable pancreatic cancer, a disease characterized by its aggressive nature and limited treatment options. (Patient enrollment and study flow are summarized in Figure 1).
- This study observed a 6-month progression free survival (PFS) rate following niraparib treatment of 41.38% (12/29 patients; 95% CI, 4.70%-100%), median PFS of 4.4 months (CI, 3.6-6.5 months), and median overall survival (mOS) of 10.3 months (CI, 7.6-15.9 months). (Kaplan-Meier estimates of progression-free survival and overall survival are presented in Figure 2).
- Differential responses in patient subpopulations harboring germline BRCA1/2 and non-BRCA1/2 mutations were also identified, offering a potential avenue for targeted therapy. (Distribution of DDR gene alterations is shown in Figure 3).

## Trial information

**Table:** 

**TRIAL INFORMATION**	
**Disease**	Niraparib in advanced pancreatic cancer with DNA repair defects (NCT03553004)
**Stage of disease/treatment**	IV
**Prior therapy**	Previous standard chemotherapy
**Type of study**	Phase II
**Primary endpoints**	6-month progression free survival.
**Secondary endpoints**	Median overall survival (mOS) and disease control rate (DCR).
**Additional details of endpoints or study design**: The primary objective of the trial was to assess the antitumor efficacy of niraparib using a 6-month PFS rate, as measured using the Response Evaluation Criteria in Solid Tumors (RECIST) version 1.1. For secondary objectives, parameters including objective response rate (ORR), OS, DCR, distribution of DOR, and occurrence of adverse events were assessed. The assessments also utilized CT/MRI scans and CTCAE v5.0. Adverse events were categorized by type, grade, system organ class, and preferred term, with serious adverse events being detailed separately. Tumor evaluations were routinely performed throughout the study using CT (or MRI if CT was contraindicated). In rare instances, cytology and histology techniques were used to distinguish between partial and complete responses. PFS was defined as the period from treatment initiation to the identification of tumor progression or death. In contrast, OS was defined as the interval from the start of treatment until death. Participants who did not experience objective tumor progression or death during the evaluation period or who were lost to follow-up were excluded (Figure 1). **Statistical analysis** Simon’s minimax 2-stage design^1^ was used to establish the sample-size justification and assess the primary objective. The primary study endpoint, the 6-month PFS rate with a 95% CI, was estimated using the approach described by Koyama.^1^ Responses, based on the RECIST criteria, were classified as complete response, partial response, progressive disease, or stable disease, and tabulated accordingly. The DCR at 8 weeks was estimated as a proportion with a 95% CI. Time-to-event data points were summarized using Kaplan-Meier curves, and medians were estimated with 95% CIs (Figure 2). **Study approval** The ethics committee of the KUCC DSMC approved the study protocol. The trial was conducted in accordance with the US Code of Federal Regulations (CFR). The participants or their legal guardians provided written informed consent.	

## Drug information

**Table:** 

**DRUG INFORMATION**	
**Generic/working name**	Niraparib
**Company name**	GSK
**Drug type**	Chemotherapy
**Drug class**	PARP inhibitor
**Dose**	300 mg or 200 mg
**Route**	Oral
**Schedule of administration**	300 mg or 200 mg administered daily for 28 days. 200 mg dose was adjusted for participants with a baseline weight of <77 kg or a baseline platelet count of <150 000 µL. Notably, an escalation from two to three capsules was allowed if no treatment interruptions occurred during the first two cycles. Participants self-administered niraparib in the morning with a full glass (≥8 oz) of water or juice. Treatment continued through additional cycles until disease progression, unacceptable toxicity, or withdrawal of consent, which varied for each participant.

## Patient demographic data

**Table:** 

PATIENT CHARACTERISTICS		
	Evaluable patients	Total enrolled
	*N *= 29	*N *= 37
**Average age** (**years**)	63	63
**Race**		
** White/Caucasian**	27 (93.1%)	34 (91.9%)
** American Indian or Alaskan Native**	1 (3.45%)	1 (2.7%)
** Black/African American**	1 (3.45%)	1 (2.7%)
** Other**	0 (0%)	1 (2.7%)
**Disease status**		
** Metastatic**	22 (75.86%)	30 (81.1%)
** Locally advanced**	5 (17.24%)	5 (13.5%)
** Recurrent**	2 (6.9%)	2 (5.4%)
**Smoking status**		
** Yes**	14 (48.23%)	20 (54.1%)
** No**	15 (51.72%)	17 (45.9%)
**ECOG**		
** 0**	5 (17.24%)	7 (18.9%)
** 1**	24 (82.76%)	30 (81.1%)

Abbreviation: ECOG: The Eastern Cooperative Oncology Group Performance Status.

## Primary assessment method

**Table:** 

**EFFICACY OUTCOMES**				
Outcome	**Patients** (***n* = 29**)	95% CI	**BRCA1/2** (***n* = 14**)	**Non-BRCA** (***n* = 15**)
**6m-PFS**	41.38% (12/29)	4.70%-100 %	50%	33.33%
**mPFS**	4.4 months	3.6-6.5 months	5.2 months	4 months
**mOS**	10.3 months	7.6-15.9 months	12.1 months	10.3 months
**DCR**	72.41% (21/29)	52.76%-87.27%	64.29%	80%

Abbreviations: 6m-PFS: Six-month Progression-Free Survival; DCR: Disease Control Rate; mOS: Median Overall Survival; mPFS: Median Progression-Free Survival.

## Serious adverse events (SAEs)

**Table:** 

SERIOUS ADVERSE EVENTS	
SAFETY PROFILE	TOTAL ENROLLED (*n* = 37)
**SAE**	5 (14%)
**TEAE**	29 (78%)
**Anemia**	10 (27%)
**Nausea**	8 (22%)
**Vomiting**	8 (22%)
**Thrombocytopenia**	7 (19%)
**Fatigue**	6 (16%)
**Grade 3 and 4 events**	12 (32%)

Abbreviations: SAE, Serious Adverse Events; TEAE, Treatment-Emergent Adverse Events.

## Pharmacokinetics

**Table:** 

PHARMACOKINETICS (mean ± SD (*N*) single dose and steady-state plasma niraparib plasma concentrations observed in patients receiving niraparib 200 mg orally once daily in Cycle 1, Day 1 and Cycle 2, Day 1)			
Parameter	Cycle 1	Cycle 2	AR
**C3** (**ng/mL**)	423 ± 323 (18)	1118 ± 570 (11)	3.06 ± 1.28 (11)
**C24** (**ng/mL**)	240 ± 190 (19)	786 ± 456 (12)	4.16 ± 2.29 (11)
**Trough** (**ng/mL**)		681 ± 535 (12)	
**Method:** Blood(plasma) samples were collected at 3 hours and 24 hours post-dose following the first niraparib dose administration on Cycle 1, Day 1 as well as Cycle 2, Day 1. In addition, a trough blood sample was obtained on Cycle 2, Day 1, prior to the first niraparib oral dose administered during the treatment cycle. Plasma concentrations of niraparib were quantified by LC-MS/MS.			

**Results:** Plasma samples were obtained across treatment cycles from a total of 24 patients. In 12 patients, Cycle 1 and Cycle 2 data were obtained at the oral niraparib 200 mg once-daily dosage regimen, as summarized in the Pharmacokinetics table. The 3-hour time point was obtained as an estimate of C_max_ based on the reported T_max_ of 3-4 hours.^2^ Plasma niraparib concentrations observed at 3 hour (C3) and 24 (C24) hour following the first oral niraparib dose and at steady state were reasonably consistent with the literature. The reported C_max_ for niraparib following a single oral dose of 300 mg was 804 ng/mL.^2^ As an estimate of the accumulation ratio, the ratios of steady-state to single-dose C3 and C24 values were approximately 3-fold and 4-fold, respectively. Similarly, Yonemori et al observed a 2-fold increase in niraparib systemic exposure following 21 days of oral dosing.^3^ We observed moderate interpatient variability in plasma niraparib concentrations at the 200-mg oral dose, which is consistent with the literature.^4^

**Table:** 

Distribution of DNA damage repair (DDR) gene alteration			
Gene	Germline	Somatic	Total
**BRCA 1**	1	1	2
**BRCA2**	5	7	12
**BARD1**	1	0	1
**ATM**	2	3	5
**CHEK2**	2	4	6
**RAD51B**	–	1	1
**NBN**	1	1	2
**ARID1A**	–	1	1
**FANCA**	–	1	1
**FAM175**	–	1	1
**IDH1**	–	1	1
**IDH2**	–	1	1
**Total**	12	22	34*
**Outcome notes:**Among the 29 evaluable patients, DDR gene alterations, including germline and somatic mutations in several homologous recombinant repair genes. BRCA 1/2 gene alterations represented the largest subgroup (*n* = 14). Other mutations were identified including BARD1, ATM, CHEK2, RAD51B, FANCA, NBN, ARID1A, FAM175, IDH 1&2. *The total number of mutations (34) exceeds the number of evaluable patients (*n* = 29) because several subjects in this study presented with more than one DNA repair defect in their cancers (Figure 3).			

## Discussion

Globally, pancreatic cancer poses a significant health challenge, with an annual estimated incidence of approximately 447 665 cases.^5^ Pancreatic cancer is the third most prevalent cause of cancer-related mortality in the US for both sexes, leading to over 50 000 deaths each year, and is expected to become the 2nd leading cause of cancer-related death in the US by 2030.^6^^,^^7^ At diagnosis, as many as 50%-60% of patients already exhibit distant metastatic disease, 25%-30% have regional disease, and only 10%-15% present with localized tumors.^8^ Treatment and prognosis further depend on the resectability of the tumor; however, only 15%-20% of patients present with resectable tumors at the time of the initial diagnosis, and the mOS for patients with distant metastases ranges from 7 to 11 months following conventional therapy.^7^^,^^9–11^

**Figure 1.**
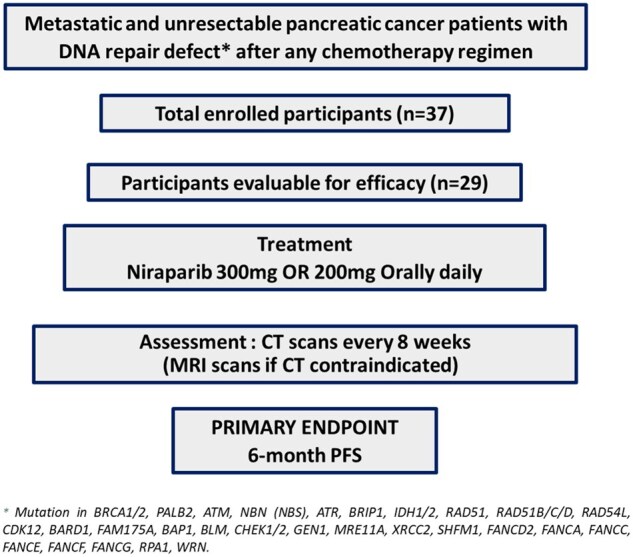
Study schema: participant flow and study profile.

**Figure 2.**
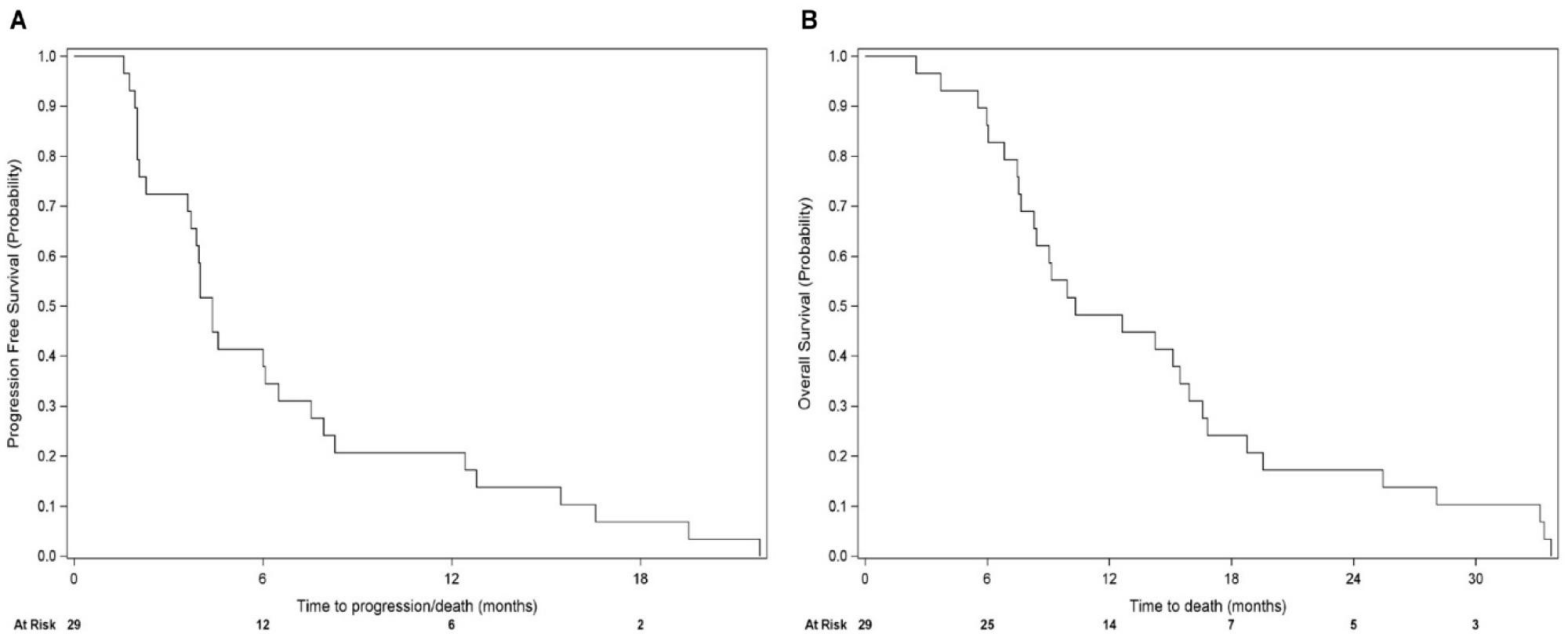
Kaplan-Meier estimates of the (A) Progression Free Survival, in months and (B) Overall Survival, in months, measured as the time span from treatment initiation with niraparib to the first observed instance of disease progression or any-cause mortality (*n* = 29, evaluable patients).

**Figure 3.**
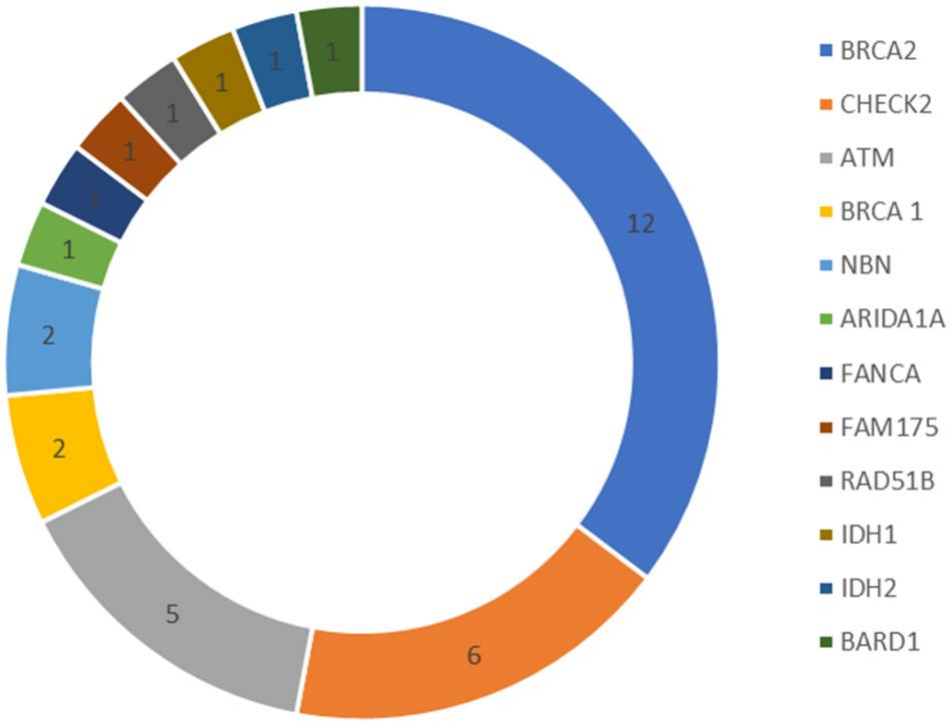
Distribution of DDR gene mutations, in number of patients (#), among metastatic or unresectable pancreatic cancer patients recruited for the study (*n* = 29, evaluable patients).

FDA-approved first-line chemotherapy regimens for advanced pancreatic cancer include gemcitabine plus nab-paclitaxel, folinic acid (leucovorin), fluorouracil (5-FU), irinotecan, and oxaliplatin (FOLFIRINOX). In patients with progressive disease following first-line chemotherapy, second-line regimens commonly used in clinical practice include 5-FU with oxaliplatin (FOLFOX) or 5-FU with irinotecan (FOLFIRI). Both of the latter treatment options reportedly result in a median overall survival (mOS) of approximately 6 months and a median progression-free survival (mPFS) of 3 months.^8^^,^^12–14^ Following first-line treatment for metastatic pancreatic cancer, many patients deteriorate quickly, and attempts to meaningfully improve survival have not been very successful. Previous studies have further indicated that this disease has a complex genomic landscape, and there is a critical need to identify specific molecular changes that define prognosis and guide therapy decisions.^15^^,^^16^ Indeed, numerous DNA repair defects result from mutations in DDR genes, such as the Fanconi anemia pathway genes BRCA1/2 (breast cancer genes 1 and 2) and PALB2 (partner and localizer of BRCA2), among others.^17–19^

Poly-(ADP-ribose) polymerases are nuclear enzymes activated by DNA single- or double-strand breaks, which then synthesize poly(ADP-ribose) [pADPr] polymers to facilitate DNA repair.^20^ Inhibiting PARP enzyme has been exploited as a therapeutic target in breast and ovarian cancers that harbor homologous recombination repair (HRR) pathway deficiencies such as BRCA1/2 or PALB2 mutations, by using a PARP inhibitor.^20^^,^^21^

The safety and efficacy of the PARP inhibitor niraparib were investigated in a phase 3 randomized, double-blind, placebo-controlled trial (ENGOT-OV16/NOVA) in patients with platinum-sensitive recurrent epithelial ovarian, fallopian tube, or primary peritoneal cancer.^22^ Based on this trial, niraparib was FDA-approved for the maintenance treatment of adult patients with advanced ovarian cancer. However, more recent data on the extent of the overall survival benefit may need further investigation.^22^^,^^23^

To date, PARP inhibitors have also demonstrated clinical activity as single agents in a subset of pancreatic cancers with BRCA1/2 or PALB2 mutations. Initial studies on olaparib showed a 23% overall response rate in BRCA mutant metastatic pancreatic cancer patients who had received prior chemotherapy.^24^ However, the phase III POLO trial later suggested that olaparib’s most significant benefit may lie in its use as a maintenance therapy, rather than as a response-inducing agent.^25^ Another PARP inhibitor, veliparib, although well-tolerated, did not achieve a confirmed response in patients with stage III/IV PDAC and a known germline BRCA1/2 or PALB2 mutation after 1-2 prior lines of treatment.^26^ Rucaparib was also studied in patients with BRCA-mutant pancreatic cancer who had been exposed to prior chemotherapy in the locally advanced or metastatic disease setting and demonstrated an objective response rate of 16%.^27^

## Therapeutic impact and clinical response

The primary study endpoint, 6-month PFS, was achieved in 41.38% of evaluable patients, with a mPFS of 4.4 months and a median overall survival (mOS) of 10.3 months. The disease control rate (DCR) at 8 weeks was observed in 72.41% of patients. These results suggest that niraparib may provide clinical benefits to a subset of patients with DDR gene defects beyond those with BRCA1/2 mutations.

Further subgroup analysis by BRCA1/2 mutation status revealed distinct responses to niraparib treatment. Patients with BRCA1/2 mutations had a higher 6-month PFS rate of 50% compared with 33.33% in the non-BRCA group. Additionally, the BRCA1/2 group had a longer mPFS of 5.2 months compared to 4.0 months in the non-BRCA group. However, patients without BRCA1/2 mutations had a shorter mOS of 10.3 months compared with 12.1 months in the BRCA1/2 group. Interestingly, the non-BRCA group had a higher DCR of 80% vs 64.29% in BRCA1/2-mutant patients. These findings suggest that, although BRCA1/2 mutations are associated with increased sensitivity to PARP inhibition, other genetic alterations in the DNA repair pathway may also influence treatment response. Given the modest survival benefit seen in the non-BRCA group, even small improvements in disease control could significantly improve patient quality of life and provide additional time for subsequent treatments.

## Comparison with existing therapeutic options

Currently, second-line treatment options for metastatic pancreatic cancer are limited. The NAPOLI-1 study demonstrated that, in patients with PDAC, combining nanoliposomal irinotecan with 5-FU improved mOS to 6.1 months compared with 4.2 months with 5-FU alone, establishing this combination as a recognized second-line treatment option for metastatic, unresectable pancreatic cancer that is unresponsive to first-line therapy.^28^ In contrast, our study suggests that niraparib may confer a survival benefit in patients with DNA repair gene mutations, with a mOS of 10.3 months. Although our study lacked a control arm, the observed mPFS and DCR suggest that niraparib might help prolong disease stabilization in selected patients.

A recent analysis by Mirza et al examined the correlation between PFS and the mutation status of 18 HRR genes in patients with recurrent ovarian cancer enrolled in the ENGOT-OV16/NOVA trial.^29^ Niraparib demonstrated PFS benefits across various mutation profiles, including somatic BRCA-mutated, BRCA wild-type with other non-BRCA HRR mutations, and those without HRR mutations, regardless of genomic instability score.^29^ These findings suggest that niraparib may have broader applications beyond BRCA/HRR-mutated ovarian cancer and support its further investigation in pancreatic cancer.

## Study limitations

Our study had a few limitations, including its single-arm design and relatively small sample size, which may affect the generalizability of our findings. The absence of a control arm limits head-to-head comparisons with standard therapies, and potential selection bias may influence the observed outcomes. Additionally, despite identifying differential responses based on BRCA1/2 mutation status, further molecular characterization of additional DNA repair pathway alterations is necessary to interpret broader predictive biomarkers for PARP inhibitor responses.

This phase II study with niraparib demonstrated clinical activity in patients with metastatic or unresectable pancreatic cancer with DDR gene mutations. This study demonstrates that PARP inhibition prolongs disease control and survival in certain patients beyond BRCA1/2-mutant pancreatic cancer, supporting the role of targeted therapy beyond conventional chemotherapeutic modalities. Given the heterogeneity of DNA repair deficiency in pancreatic cancer, further research is required to refine molecular testing and patient selection based on DDR gene alterations to optimize treatment strategies. Further studies should focus on larger, controlled trials to validate our results and investigate combination treatments that may enhance the effectiveness of niraparib.

## Data Availability

Data is available upon request.
